# Probabilistic Modeling of Microbial Metabolic Networks for Integrating Partial Quantitative Knowledge Within the Nitrogen Cycle

**DOI:** 10.3389/fmicb.2018.03298

**Published:** 2019-01-28

**Authors:** Damien Eveillard, Nicholas J. Bouskill, Damien Vintache, Julien Gras, Bess B. Ward, Jérémie Bourdon

**Affiliations:** ^1^LS2N, UMR6004 CNRS, Université de Nantes, Centrale Nantes, IMTA, Nantes, France; ^2^Research Federation (FR2022) Tara Oceans GO-SEE, Paris, France; ^3^Climate and Ecosystem Sciences Division, Lawrence Berkeley National Laboratory, Berkeley, CA, United States; ^4^Geoscience Department, Princeton University, Princeton, NJ, United States

**Keywords:** modeling, microbial ecology, ammonia oxidizing bacteria, probabilistic simulation, nitrogen

## Abstract

Understanding the interactions between microbial communities and their environment sufficiently to predict diversity on the basis of physicochemical parameters is a fundamental pursuit of microbial ecology that still eludes us. However, modeling microbial communities is problematic, because (i) communities are complex, (ii) most descriptions are qualitative, and (iii) quantitative understanding of the way communities interact with their surroundings remains incomplete. One approach to overcoming such complications is the integration of partial qualitative and quantitative descriptions into more complex networks. Here we outline the development of a probabilistic framework, based on Event Transition Graph (ETG) theory, to predict microbial community structure across observed chemical data. Using reverse engineering, we derive probabilities from the ETG that accurately represent observations from experiments and predict putative constraints on communities within dynamic environments. These predictions can feedback into the future development of field experiments by emphasizing the most important functional reactions, and associated microbial strains, required to characterize microbial ecosystems.

## 1. Introduction

Recent advances in molecular biology and computational biology have transformed approaches to characterize microbial communities (Segata et al., 2013; Waldor et al., 2015), prompting the emergence of microbial systems ecology. This field tackles complex ecological questions by coupling observational (e.g., molecular and geochemical) data with new computational techniques (Raes and Bork, 2008; Klitgord and Segrè, 2011; Zelezniak et al., 2015). Advances in bioinformatics and computational biology have allowed analysis of next-generation sequencing technologies to qualitatively describe microbial communities by emphasizing “*who is there and who is not”* (Raes et al., 2011). However, among the most significant challenges in microbial systems ecology is the ability to quantitatively predict microbial community composition and function, by combining molecular data and quantitative physicochemical data. Theoretically, this challenge necessitates the consideration of both measurements (e.g., community composition or associated geochemistry) alongside an uncertainty analysis associated with these measurements. However, such a coupling is still elusive in predictive modeling (see Mouquet et al., 2015; Delahaye et al., 2017 for review, or Legay et al., 2010 for a similar question in the broad context of Computer Sciences). Previous applications in ecology (e.g., Jabot and Chave, 2011; Marion et al., 2012), promote the use of advanced computational approaches to integrate statistical analysis into a mechanistic modeling framework, but both concepts of determinism and randomness are still usually considered as independent (Anand et al., 2010).

Among the techniques that integrate uncertainties, the Bayesian network is a probabilistic graph model that represents the biological compound interactions via a directed acyclic graph (Friedman et al., 2000). However, the Bayesian network is not able to take into account the feedback loops necessary to represent the accumulation of quantities over time (e.g., the abundance of mico-organisms or concentrations), such as is necessary to depict general biological dynamical behaviors. For this purpose, it would be preferable to use an extension of Bayesian networks: dynamical Bayesian networks. These dynamic networks consist of the repetition of elementary Bayesian networks, as previously defined, linked together in order to abstract dynamical effects, including feedback loops. Nevertheless, despite being of practical interest, such a combination of networks drastically increases model complexity. Such an extension is not always appropriate to model mechanistic behaviors, such as trophic interactions. By proposing Probabilistic Boolean Networks (PBN), Shmulevich et al. (2002) propose a new probabilistic approach, that is not Bayesian, to model mechanistic behaviors. PBNs combine the expressibility of Boolean networks to describe dynamical deterministic behaviors and uncertainty via the use of probability (see Li et al., 2007 for a more complete comparison between PBNs and dynamical Bayesian networks in the context of gene regulatory circuit modeling). Overall, PBN represents a general probabilistic modeling framework that combines deterministic modeling and uncertainties. PBN offers plenty of applications in the context of biological networks, with a strong emphasis on qualitative modelings. Nevertheless, PBN does not permit quantitative modeling. For this purpose, Bourdon et al. (2011) proposed a complementary approach of PBN modeling; still not Bayesian, called Event Transition Graph (ETG). This approach combines Boolean modeling and probabilistic approaches but integrates descriptive mechanistic measurements alongside more quantitative knowledge that is required to depict ecological properties, usually attributable to continuous variables.

**Figure 1:**
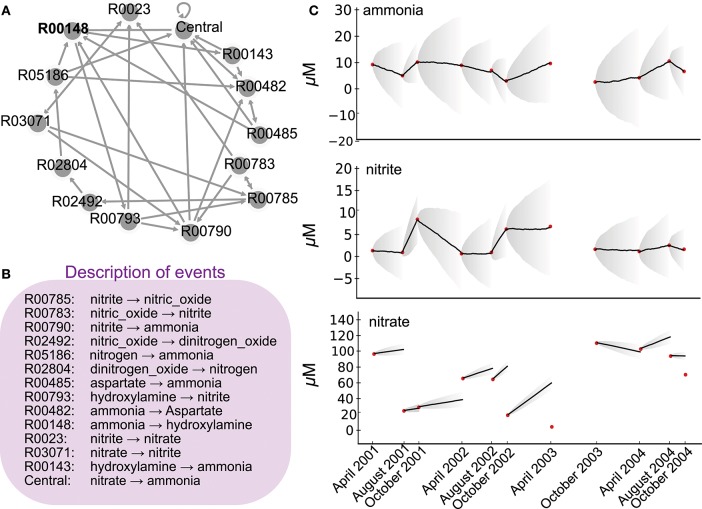
Network of the nitrogen cycle and its probabilistic simulation. **(A)** represents the nitrogen cycle where nodes are reactions as described in KEGG, and edges putative transitions between reactions when a product of a reaction is a substrate of another. **(B)** depicts in the simplistic metabolic effect asociated to each reaction involved in **(A)**. **(C)** depicts 11 time point nutrient concentrations (red dots) as described in Bouskill et al. (2011) station CB100 in Chesapeake Bay, as well as nine probabilistic simulations of the ETG model trained on ammonia and nitrite concentrations between 2001 and 2004. Corresponding probabilities are depicted in Figure 2. Bold lines depict averages of quantities over 10 simulations, whereas gray areas show the corresponding standard deviations.

ETG was originally developed to model multi-scale systems and Bourdon et al. (2011) used it to determine the impact of *E. coli* gene regulatory networks on intracellular protein concentrations under diverse growth conditions (Ropers et al., 2006). Unlike traditional biological modeling techniques (e.g., ordinary differential equation approaches where all processes are equivalent), ETG classifies the order of biological events, such as gene transcription, and transitions from one state to another via a set of probabilities such that the succession of states accurately reproduces experimental observations. Such a classification of biological events, being controlled only by probabilities, avoids the need for kinetic parameterization, which is usually unknown for microbial ecosystems, but rather advocates for the addition of uncertainties to a deterministic schema. In other words, required inputs for ETG modeling are (i) the chronological and mechanistic descriptions of biological events (i.e., metabolic reactions) and their potential connections (e.g., auxotrophy), and (ii) a quantitative behavior to reproduce (e.g., the trajectory of functional groups under fluctuating environmental conditions, or time series of quantities as presented in Figure 1). As a result, ETG will learn parameters from quantity variations while considering uncertainties. In this purpose, ETG weighs the transitions between discrete events by probabilities which reproduce, on average, the quantitative behaviors observed in nature. As a result, ETG could mimic a dynamical quantitative system by integrating, in a non-deterministic manner, several mechanistic descriptions within a probabilistic framework. The main insights gleaned from this approach can bring further understanding and prediction of the temporal succession of community assemblages (Fuhrman et al., 2006; Bouskill et al., 2011). In particular, this approach could relate key microbial functional guilds to changes in the metabolites consumed or produced across gradients in co-occurring and interacting environmental variables.

Herein, we briefly describe the ETG modeling approach and the associated requirements for running the programs. We will then demonstrate the application of ETG within the context of microbial ecology for the first time. We focus here on the nitrogen cycle. Beyond the intrinsic importance of nitrogen for biological systems, its cycling results from versatile redox chemical reactions. Combined together, these reactions promote complex biogeochemical transformations and structure microbial communities. From a modeling viewpoint, the nitrogen cycle presents three features that make it a promising candidate for new quantitative modelings. First, and despite recent studies uncovering new reactions and pathways (Kuypers et al., 2018), nitrogen metabolic pathways are well-understood and therefore constitute a metabolic map that provides a stable and mechanistic description of the biological processes involved (Kanehisa and Goto, 2000). This map represents a set of biological events that can be quantitatively described. Second, because of recent technological advances, especially in biogeochemistry and isotopic studies, the main processes involved in nitrogen transformation (e.g., nitrogen fixation, nitrification, denitrification) can also be depicted through quantitative rate measurements, which provide an overall ecosystem behavior. These rates are ETG goals to be reproduced by the trained model. Finally, high-throughput sequencing technologies provide greater insight into about the ecology of the microbial functional guilds playing an important role in the nitrogen cycle, in particular, the organisms responsible for different redox reactions and their putative interactions (see Jewell et al., 2016 for an illustration).

## 2. Materials and Methods

### 2.1. Event Transition Graph Modeling: Data and Biological Knowledge Formatting

ETG requires expert biological knowledge be formalized as a graph. Experimental knowledge will be then incorporated into the model via a learning procedure that weights the edges of this graph.

#### 2.1.1. Network or Graph of Interactions

The first input into ETG modeling is a list of biological events as well as the consequences of these events. For the sake of illustration, when representing the nitrogen cycle, the events are reactions (e.g., nitrification, denitrification, etc.) and their consequences are the respective production and consumption of metabolites (e.g., NH_4_^+^ NO_3_^−^). This knowledge is a mechanistic description of an event and is necessary to estimate the “*cost,”* or effect when one event occurs over another. Here we derive a nitrogen network composed of a hypothetical series of reactions (i.e., fixation, nitrification, denitrification and anammox), as laid out in the Kyoto Encyclopedia of Genes and Genomes (KEGG) database map00919 (Kanehisa and Goto, 2000), without assigning taxonomy to the microorganisms that mediate these reactions. For reducing the complexity of the nitrogen cycle, for each reaction, we considered major metabolites and neglected co-factors. Reversible reactions were decomposed into two opposite irreversible reactions. We then removed reactions that propose similar metabolic mechanistic transformations. After removing duplicated reactions, this set of reactions, called sequential biological events, consists of 14 reactions (see Supplementary Material for technical details and required format).

Concomitantly, as an additional modeling input, interactions between events take the form of a graph that links reactions (i.e., nodes of the graph) when the product of one reaction becomes the substrate for another reaction (directed edge). Thus, the above 14 reactions result in a graph of 14 nodes and 32 edges (see Supplementary Material for a technical graph description) and illustrated in Figure 1A. The combination of the graph of events and the effect of each event represents a mechanistic description of the modeled biological system. The effect of each event is additive to simulate an effect of stoichiometry, but, as proposed in Bourdon et al. (2011), a multiplicative effect could also be used to represent exponential behaviors. Notice herein, for closing the system (i.e., no transition must point out), the hypothetical model considers a central reaction. It depicts an artificial reaction that points toward reactions linked to the nitrogen cycle but involved in other metabolic pathways, such as carbon or phosphate, as mentioned in the KEGG database. For consistency with other reaction descriptions, its mechanistic description considers only major nitrogen metabolites.

#### 2.1.2. Initial Costs

In addition to the overall definition of an event (i.e., reactions and product/substrate definition) and description of the interactions within events (through the construction of a graph), the cost of considering one event over another must also be defined. As a mechanistic description, each event consumes and produces compounds, which will point to the cost of using events. For instance, each reaction within the nitrogen cycle can be described by its stoichiometry (i.e., –1 for a metabolite consumption and + 1 for a metabolite production). However, when randomly crossed, the graph could promote an artificial increase or decrease of a given compound, solely due to the graph topology and chemical stoichiometry. Such a result would not represent a correct output of the modeling approach, but rather a prospective flaw. To avoid this, one must compute the cost (denoted initial cost) for all compounds for each event, that is not the stoichiometry *per se*. This cost is necessary to maintain every compound at a stationary amount when every transition is equiprobable (i.e., steady states). For each compound, this initial cost will be assigned to events that do not mention them explicitly. For instance, for all reactions that do not consider ammonia, nitrite or nitrate as metabolites, one must compute a cost for these metabolites. Thus, following a computational procedure described in Supplementary Material, –1.5, –1.00, and –0.25 are the costs related to these metabolites (resp. ammonia, nitrite, or nitrate) when not explicitly mentioned in their stoichiometry. The costs are not necessary constrained by units and costs of different units could be considered simultaneously. Biologically, the negative cost could be interpreted by a putative dispersal of metabolites when not explicitly produced or consumed by a metabolic reaction.

#### 2.1.3. Formating the Quantitative Data as Training Dataset

ETG modeling estimates probabilities associated with interactions between events (herein reactions) such that the succession of events reproduce quantitative experimental data. For illustration, we use chemical variables from Bouskill et al. (2011), which describes a time series of ammonia, nitrite, and nitrate (see Table 1). In order to fit such quantitative experimental data with ETG, one must transform quantitative variations as rates, which necessitates the assignment of a time-step. For instance, when considering a time-step of two hours, a variation from 8.4 to 4.2 μM of ammonia between April and August 2001 requires 1,476 time-steps (123 days x 12), representing an overall variation rate of:

$$\begin{array}{r} \text{rat}{\text{e}}_{\text{N}{\text{H}}_{3}}=\frac{4.2-8.4}{1476}\approx -0.0028455 \end{array}$$

**Table 1:** Dissolved inorganic nitrogen concentrations (μM) over the time-course of dataset from sampling station CB100 surface as presented in Bouskill et al. (2011).

**Time course samples**	**Ammonia (μM)**	**Nitrite (μM)**	**Nitrate (μM)**
April 2001	8.4	0.8	88.7
August 2001	4.2	0.4	19.9
October 2001	9.3	7.9	24.2
April 2002	8.1	0.1	59.1
August 2002	6.2	0.4	11
October 2002	2.2	5.7	19.3
April 2003	6.3	0.5	76.8
October 2003	1.8	1.1	101.9
April 2004	3.4	0.6	94.7
August 2004	9.7	2	86.2
October 2004	5.8	1.1	63.7

Experimental variation in rates for each season (from April to August, from August to October, and from October to April) for the years 2001, 2002, 2003, and 2004, and for each nutrient was thus estimated from Table 1. These rates are the training data and represent the quantitative variations that must be reproduced by the probabilistic modeling once parameterized. As detailed below, ETG will learn probabilities to reproduce these quantitative rates. They imply to consider predefined time-steps, but also allow to not constraints the cost units or even considering the costs of distinct units simultaneously.

### 2.2. Probability Estimation and Probabilistic Simulations

**Figure 2:**
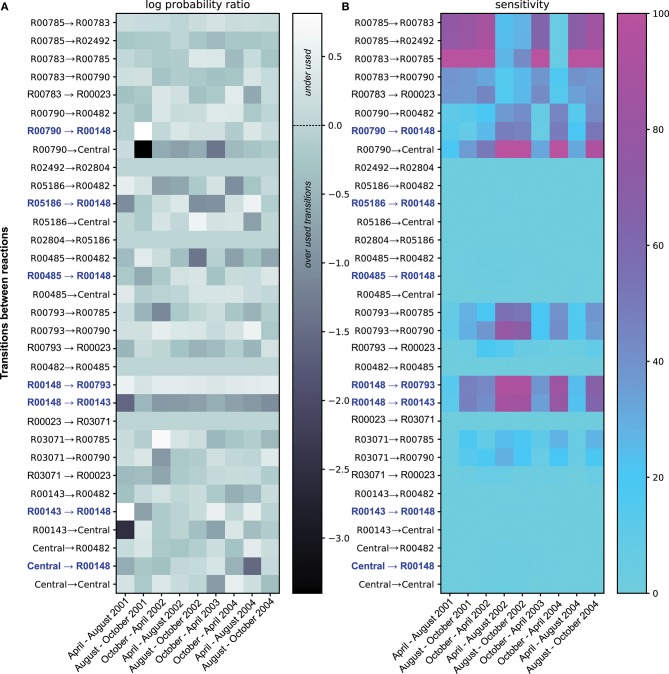
Summary of the ETG model Probabilities and Sensitivities trained on ammonia and nitrite concentrations. Panel **(A)** shows the log ratio of computed probabilities over probabilities of each transition under the equiprobability assumption. Transitions illustrated in light gray show probabilities in the equiprobability assumption. The dark gray color represents transitions with probabilities lower than those computed under the equiprobability assumption, whereas lighter colors are transitions with higher probabilities. Transitions colored in blue depict either transition pointing toward or coming from R00148; reaction catalyzed by the product of *amo* gene. Panel **(B)** gives sensitivity values for each transition. Cyan transitions are not sensitive, whereas purple transitions are the most sensitive, i.e., the probability values cannot change without altering the overall predictive accuracy.

Once the ETG model considers (i) a set of events and their putative interactions (section 2.1.1); (ii) a cost for each event (section, one (iii) a quantitative rate that depicts an experimentally observed quantitative variation impacted by at least one event (section 2.1.3), one seeks then to learn probabilities to prioritize interactions between events in order to reproduce the above computed rates as they resume the environmental conditions to reproduce. The overall parameterized model will herein reproduce variations of ammonia, nitrite, and nitrate by weighting the succession of metabolic reactions (e.g., the cost of consuming or producing a given compound resuming a reaction). An optimization process (see technical details in Bourdon et al., 2011) will compute sets of probabilities for all transitions between each sample within a time series. ETG applied on the toy model of the nitrogen cycle will thus compute nine distinct sets of probabilities that reproduce the rate of variation of ammonia, nitrite and nitrate over four years (i.e., number of columns in Figure 2). It is important to notice herein that searching for optimal probability values is performed by a local search method. Local search methods are sensitive to sub-optimal solutions. Despite the use of a metaheuristic (i.e., Tabu search; Glover, 1986) that memorizes visited solutions, finding the best solution is complex (NP-hard), which could be prejudicial for larger complex models. However, from a practical viewpoint, models with 15 nodes and 30 edges remain realistic on a personal computer.

Along with probability estimates for transitions between each event, a sensitivity score (*S*), expressed in percentage, was also computed. The *S* score associated with a transition expresses the fact that the Euclidean distance between the expected rates (goals from section2.1.3) and their predictions is modified by *S* % when its probability value is changed by 1%. Such a sensitivity score permits ranking the transitions according to their respective sensitivities (i.e., a high sensitivity transition implies higher constraints on its corresponding probability value). In practice, sensitivities between two-time points depict in Figure 2B are the mean sensitivities of 100 optimal probability estimations that reproduce ammonia and nitrate experimental variations.

**Figure 3:**
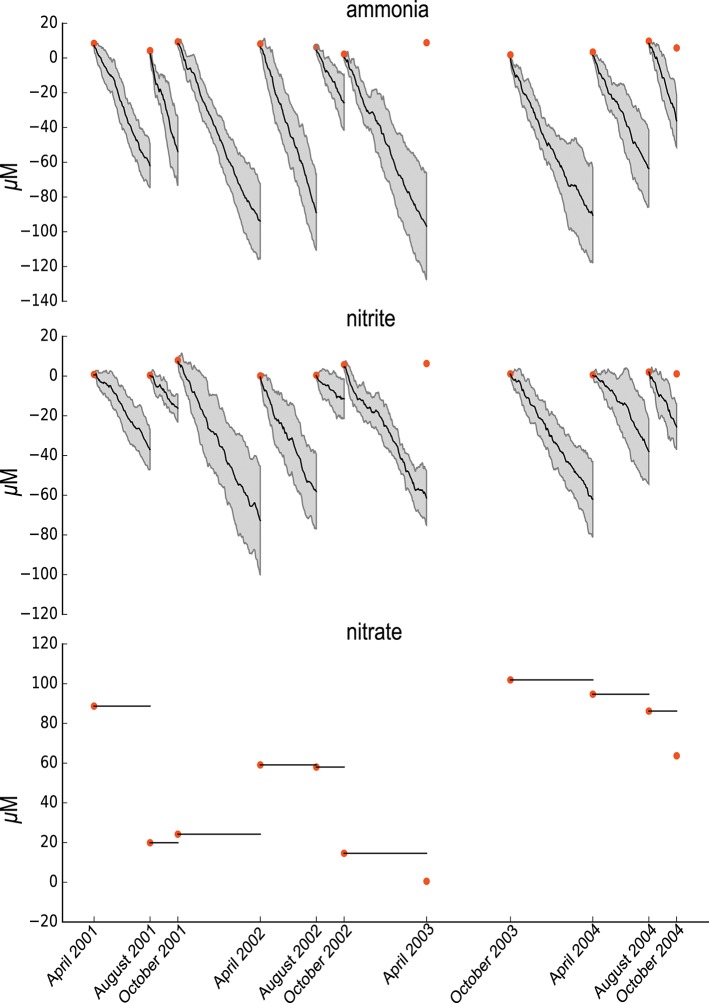
Summary of the random ETG model Probabilities and Sensitivities trained on ammonia and nitrites concentrations.

Following the training protocol, a Markov Chain simulation algorithm allows to simulate the variation of quantities over time. As input, the simulation considers (i) initial quantities (i.e., red dots in Figure 1C or Figure 3) and cost (i.e., production and consumption of metabolites when a reaction occurs) and (ii) above learned probabilities that describe respectively in the stochastic paradigm: (i) the reaction constants and (ii) the random number generator. Altogether, these features describe a stochastic system for which a Monte Carlo step determines the reaction that occurs at each time interval. At a given time, the probability of choosing a given reaction is, therefore, the compromise between the costs, that describe how the molecules evolve for a given event, and the duration of an event (time-step) that one fixed in our study to 2 h. In the context of this study, for each time point (i.e., transition between two successive red dots), the simulation protocol will perform 10 independent stochastic simulations. Figure 1C and Figure 3 represents average of simulated quantities over time (i.e., bold line) as well as associated standard deviations (i.e., gray areas). Notice that such stochastic simulations of ETG are closely related to simulations performed by the Gillespie algorithm in its asymptotic regime as shown in Picard et al. (2015).

However, there is no notion of atoms in the ETG simulation. Indeed, our simulation process differs from the Gillespie algorithms in the sense that the probabilities of reaching an event are supposed to be constant. In the Gillespie algorithm, there is a significant compromise between the number of molecules of a particular species and the volume of the cell. In our simulation method, the compromise is between the costs, that describe how the molecules evolve for a given event, and the duration of an event (time-step) that one fixed in our study to 2 h.

### 2.3. POGG: A Software for Event Transition Graph Modeling

The Event Transition Graph (ETG) modeling was performed via a Python package called POGG. This package is the first python implementation of the ETG modeling, as proposed in Bourdon et al. (2011). POGG package can be downloaded here, including a docker container. See Supplementary Material for technical details and a complementary script that replicates all enclosed results, including visualization.

## 3. Results

### 3.1. Probability Estimate for Simulating the Nitrogen Cycle

Ammonia oxidizing organisms (AOO) mediate the rate-limiting step of nitrification (i.e., NH_3_ → NO_2_), a rate-limiting step in the Nitrogen cycle (Ward et al., 2007; Bouskill et al., 2011, 2012). Analyzing the dynamic of the nitrogen cycle in a given ecosystem and, in particular, the impact of this reaction allows to evaluate the putative role of these organisms in the same ecosystem. Herein, we describe a schematic nitrogen cycle within a unique ETG of quantitative chemical variables and simulate the corresponding metabolic network to assert the role of the typical reaction of AOO. Following an automatic extraction from KEGG database (Kanehisa and Goto, 2000), ETG that covers the whole set of reactions associated to the nitrogen pathways represents 41 nodes and 67 edges. In the present case, for the sake of clarity, the graph is pruned to 14 nodes and 32 edges (Figure 1A). The ETG graph describes transitions across biochemical pathways with each individual reaction, or event for sake of generalization, having an effect on downstream processes (e.g., each event may produce or consume a compound according to a stoichiometrically balanced reaction equation). In the present case, one substrate can be consumed by several other reactions, which results in multiple edges per node. The ETG modeling considers the graph that resumes stoichiometry constraints but also computes a complimentary cost for each event for the sake of dynamic behavior. The cost of each event is thus parameterized in order to maintain stable concentrations for each product when transitions of the network are equiprobable (i.e., the null assumption).

To estimate probabilities between reactions and train the ETG, we used an existing environmental dataset representing variations in Chesapeake Bay ammonia, nitrite, and nitrate concentrations (μM) between 2001 and 2004 (Bouskill et al., 2011). The optimization process emphasized a set of probabilities that reproduce observed variations in ammonia and nitrate despite the use of a simple graph that strongly reduce the nitrogen cycle. To test our model, we simulated variations in chemical factors using a Markov Chain simulation algorithm parameterized with computed probabilities, and compared the predictions with the available time series data (see Figure 1B). The model accurately replicates ammonia and nitrite chemical variables over the period between 2002 and 2004, but fails to reproduce the observed nitrate dynamics. This point indicates the limit of the simple mechanistic description of the nitrogen cycle without considering external physical forcing or the need for further modeling extensions that could integrate recently discovered new reactions or pathways (Kuypers et al., 2018), especially to integrate nitrate concentration variations. Please notice also that no set of probabilities were able to replicate properly variations of concentration between April 2003 and October 2003, indicating the sensitivity of our probabilistic modeling to either the time-step or natural perturbations. Indeed, this inability to simulate this particular time slot could be related to the hurricane Isabel, strongest hurricane in the Atlantic in 2003, that hit the Chesapeake Bay just before sampling. Such a strong perturbation modified the AOO assemblage (Bouskill et al., 2011) which could affect, as well, the succession of metabolic reactions compared to regular conditions.

Beyond the probabilistic simulations, the analysis of probabilities between reactions (*i.e*. likelihoods of transitions between two reactions) are of interest. Figure 2A shows the log ratio of computed probabilities over probabilities under the equiprobability assumption, for each transition over the time period. Over the four years, some transitions between reactions show probability values that are similar, or close, to the values corresponding to the equiprobability assumption (i.e., light gray in Figure 2A). Herein, the graph topology remains the main factor to explain the use of these transitions. However, other reaction transitions show probability values very divergent than those obtained under the equiprobability assumption. Transitions depicted in white are underused, whereas those colored in darker gray are overused compared to an equiprobable use of transitions. Among the overused reaction transitions, some transitions show strong variabilities of probability values over the 4 years, whereas others are more constantly overused. In particular, the transition between ammonia-monooxygenase (R00148) and the hydroxylamine oxidoreductase reaction (R00143) is of great interest. This transition is necessary and continuously over-used over the four years, which implies a reversible transformation between ammonia and hydroxylamine. This relationship could be explain by their complementing functions that are necessary together for full oxidation of ammonium to regenerate electrons.

Concomitantly, the transition between R00148 and R00143 indicates a small efficiency of transforming ammonia into nitrite. Combined, both results emphasized the need to constrain fluxes between ammonia to hydroxylamine and back to replicate the variation of quantities; fluxes in which, among others, AOO could be involved by carrying the *amo* gene.

Figure 2B shows the sensitivity analysis of the model by emphasizing the most constrained transitions; i.e., transitions for which the probability values cannot change without altering the training efficiency. These transitions are the most constrained when the system must replicate the quantitative variations used during the training process. Logically, identification of the most sensitive transitions extracts the transition from R00148 and R00785, as these events are necessary to mediate NH_3_ and NO_2_ transformations that are required to reproduce training conditions in Figure 1B. From a biological viewpoint, this result confirms the interest in studying ammonia-oxidizing bacteria and nitrite-oxidizing bacteria, drivers of these transitions, in the Chesapeake ecosystem. However, it also emphasizes the need to foster this theoretical insight in the broader study of the global metabolic profiles of the Bay ecosystem for a comprehensive understanding of the whole biological processes carried out by the microbial communities.

Additionally, the model shows interactions of these reactions with others that are also of interest. Dissolved inorganic nitrogen concentration variations (see Figure 1B and Table 1) temporally influence the sensitivity of the reactions involved in nitrification, ammonification, and denitrification. The pressure to reproduce given dissolved inorganic nitrogen variations constraints as well the amount of other substrates and their use via reactions that do not use ammonia or nitrite *per se*. This interdependency explains patterns of sensitivities that one can not discern in Figure 2A. Interestingly, despite the heterogeneous nature of the chemical measurements, the sensitivity analysis emphasizes antagonistic patterns of two sets of reactions. On one side, a set of transitions between R00790 to central, R00793 to R00790, R00148 to R00793, and R00148 to R00143 depict approximately the ammonification and ammonia oxidation subsystem. On the other side, R00785 to R00783, R00785 to R02492, and R00783 to R00785 describe the denitrification subsystem. Overall, the sensitivity analysis emphasizes both subsystems as antagonistic over time. It is worth noting that sensitive transitions and corresponding subsystems may indicate potential constraints (or biochemical trade-offs) on organisms mediating the targeted reactions, which might be related to selective pressures at an environmental level. These pressures occur antagonistically on both denitrification and ammonification/ammonia oxidation subsystems and are the results of arbitrary rules within the ETG model that represent interactions between reactions.

### 3.2. Learning on a Random Network

The general criticism about probabilistic models concerns their use as a statistical protocol that reproduces observed data with no biological specificity. Contrary to other probabilistic modelings, ETG considers a mechanistic interpretation of the systems via the use of a graph of events. The use of a description of events allows specifying the model to perform a given (biological) behavior and to test it regarding experimental data. For an illustration of the interest of ETG specificity, we propose to build a counterexample by randomizing the model and training it on the same dataset.

The randomized model consists of building a graph similar to the nitrogen cycle graph for which all edges have been shuffled by permutation. The randomized model is then similar to the ETG nitrogen cycle model regarding the numbers of nodes and edges. We then applied a similar modeling and training procedure to that described above. As pictured in Figure 3, the randomized model, that is mis-specified, is unable to predict the variabilities in ammonia or nitrite. Indeed, no simulations permitted accurate depiction of the ammonia and nitrites experimental data. Furthermore, nitrate quantities remain constant over time, which means that the trained model could not predict changes in nitrate which highlights the need for further details (i.e., specifications) about nitrates.

## 4. Discussion

The goal of this study is to demonstrate the interest of the ETG modeling framework. In this purpose, one uses a reduction of the nitrogen metabolic network. From the biological viewpoint, despite partial promising outcomes, several modeling results do not reproduce the experiments. First, the probabilistic model does not accurately simulate the variation of nitrate, while reproducing ammonia and nitrite quantity variations. Second, and not presented in this study, the model is not able to simulate ammonia and nitrite quantities as taken from anaerobic samples (Bouskill et al., 2011), which indicates either the need to consider further details regarding oxygen, additional constraints about nitrate, or the general drawback of reducing the nitrogen cycle to its sole major metabolites. However, we consider that emphasizing these inconsistencies is of interest for further modelings that would better specify missing biological events.

Unlike other probabilistic modelings, ETG modeling is less plastic. The modeling requires a qualitative description of biological events that take place to reproduce quantitative biological data. The qualitative specification constrains the model by describing all putative biological behaviors (i.e., the succession of events and their effects). Among them, once learned, probabilities allow considering a few to reproduce a given quantitative behavior. Compared to general Bayesian modelings, this combination of qualitative and quantitative knowledge makes our probabilistic modeling sensitive to mechanistic descriptions that take the form of scrupulous accumulations or consumptions of quantities over time (Picard et al., 2017). However, this characteristic drastically increases its computational complexity compared to other state-of-the-art probabilistic modelings that are less biologically specified.

This ETG framework is ideal for investigating the dynamic and transient nature of microbial ecosystems when few quantitative knowledge is available. ETG does not begin with an assumption of a community at steady state, unlike Flux Balance Analysis techniques to model metabolic networks (see Perez-Garcia et al., 2016 for review, for metabolic modeling of microbial ecosystems see Zomorrodi and Maranas, 2012; Budinich et al., 2017). However, ETG modeling assume that observed quantitative variation are the result of an asymptotic behavior of the probabilistic model. Studying transient behaviors is advantageous to model the effect of microbial communities because, (i) *in situ* measurements are unlikely to be made at equilibrium, and (ii) most studies focus on community changes, which is itself a transient behavior. Modeling such transient behaviors is the aim of state-of-the-art continuous modelings. However, contrary to these techniques, ETG promotes the use of simple mechanistic descriptions that do not consider kinetic parameters and initial conditions *per se* to simulate quantity variations over time, but respectively the probabilistic combination of simple additive laws and quantitative rates to reproduce.

Applied to the metabolic network modeling, ETG emphasizes the biochemical constraints (i.e., the transition between reactions) necessary to satisfy for reproducing variations of quantities emerging from the biological system. One advocates that these constraints could impact as well the microbial communities that are providing the constrained metabolic reactions. To validate this assumption, one must consider further biological knowledge such as a systematic description of the microbial ecosystem over time. For instance via 16S rRNA sequencing, one could associate the patterns of microbial diversity with the metabolic constraints as highlighted by the ETG (i.e., sensitivities) and further compared them to co-occurrence patterns (Cram et al., 2015).

Similarly, the same dynamical property should be a benefit to decipher subsets of metabolites that are of interest in a given ecosystem. In this purpose, one must associate this result with genomic descriptions of prokaryotic organisms, for instance via metatranscriptomic or metagenomic studies. Such association between modeling outcomes and meta-omics knowledge could drive future definitions of the keystone species (i.e., *who is carrying the essential metabolism*) or the analysis of exchanges of interest between microbial strains (Borenstein et al., 2008; Bordron et al., 2016).

Finally, this study considered an hypothetical metabolic network as a qualitative description, but ETG is a modeling paradigm that could consider other qualitative descriptions of the microbial ecosystem such as co-occurrence networks (Patel et al., 2010; Faust and Raes, 2012; Fuhrman et al., 2015; Guidi et al., 2016) or gene associations (Coles et al., 2017). The integration of these new abtractions with partial quantitative knowledge such as chemical parameters or metabolomic within the ETG framework could be of interest to refine biogeochemistry models while considering the microbial complexity as highlighted via recent omics descriptions.

## Author Contributions

DE, NJB, BW, and JB designed the study. DE, DV, JG, and JB performed the study. DE and NJB analyzed the data. DE, NJB, DV, BW, and JB wrote the paper.

### Conflict of Interest Statement

The authors declare that the research was conducted in the absence of any commercial or financial relationships that could be construed as a potential conflict of interest.
