# Supplementary material for: Probabilistic Modeling of Microbial Metabolic Networks for Integrating Partial Quantitative Knowledge Within the Nitrogen Cycle
Source: Front Microbiol. 2019 Jan 28;9:3298. doi: 10.3389/fmicb.2018.03298 (PMC6360161; doi:10.3389/fmicb.2018.03298)
Supplement: Supplementary file 1 [file Image_1.pdf]

Supplementary Material: Probabilistic modeling of microbial networks for integrating partial quantitative knowledge within the nitrogen cycle

Damien Eveillard*, Nicholas J. Bouskill, Damien Vintache, Julien Gras, Bess B. Ward and Jérémie Bourdon

*Correspondence:
Damien Eveillard:
damien.eveillard@univ-nantes.fr

POGG SOFTWARE REQUIREMENTS AND INSTALLATION

The POGG software requirements and installation details are available at `https://gitlab.univ-nantes.fr/pogg/pogg`

PROTOCOL TO BUILD AN ETG MODEL

All the following steps are necessary to reproduce the manuscript results, but also could be used as a guidance for any ETG model.

Design of Event and their interactions

The ETG modeling considers (i) the set of biological events such as metabolic reactions and (ii) the connections between the events. These two knowledge must be formalized separately via two distinct files. For the sake of illustration, two below complementary files are necessary to replicate the companion study, and could be considered as an application guidance for other modeling studies:

- `ETG_Nitrogen.txt` as a description of biochemical reactions involved in the biological system, (denoted 'event' in a more general context). It describes the set of metabolic reactions related to the nitrogen pathway as stored in KEGG without considering taxonomy. This set of events consists in 14 reactions after removing duplicated ones. Complementary, one must describe consequences of these events. Herein, they are consumptions of substrates and productions of products. Such knowledge is necessary to estimate the cost when an event occurs rather than another.
- `ETG_Topologique_Nitrogen_squelette.dot` as a description of the connection between the nitrogen reactions within the system. It describes a graph that represents the above nitrogen cycle metabolic reactions (*i.e.*, 14 nodes after removing duplicated reactions) and their interaction (*i.e.*, 32 edges) in a dot format. For illustration, Figure 1 represents the corresponding reaction network, as proposed by graphviz software.

Setting the initial costs

These initial costs will be computed by the command `Stoichiometry.py` using both the graph in `ETG_Topologique_Nitrogen_squelette.dot` (encoded as a transition matrix) and the list of reactions as stored in `ETG_Nitrogen.txt`:

```
python -m pypogg.Stoichiometry -sf
        ETG_Topologique_Nitrogen_squelette.dot -rf ETG_Nitrogen.txt
```
(1)

This instruction will compute the initial cost for each compound involved in the model. In the sequel, we will focus on three compounds of interest: ammonia, nitrite and nitrate for which one obtains the following initial costs:

```
cost_ammonia = -1.50
cost_nitrite = -1.00
cost_nitrate = -0.25
```

Setting the experimental knowledge

Physico-chemical variables from Table 1 must be changed as rates. Within this script, an arbitrary time-step could be defined. Running:

```
python -m scripts.goals
```
(2)

allows to compute a variation rate for each nutrient from the above raw data for each season (from April to August, from August to October and from October to April) for the years 2001, 2002, 2003 and 2004, and for each nutrients. The output file of `scripts.goals` must be in the following format to train the probabilistic modeling as defined in `poggConfigFile_season_timestep.txt`:

```
Time Step 2h Ammonia April2001 August2001 -0.00284552845528 1476
Time Step 2h Nitrite April2001 August2001 -0.000271002710027 1476
Time Step 2h Nitrate April2001 August2001 -0.0466124661247 1476
Time Step 2h Ammonia August2001 October2001 0.00696721311475 732
Time Step 2h Nitrite August2001 October2001 0.0102459016393 732
...
```

LEARNING PROBABILITIES

Once the ETG model defined, one seeks to learn probabilities to prioritize interactions between events in a way that the overall parameterized model will reproduce compound variations similar to experiments. Such an optimization process detailed in Bourdon et al. (2011) will be performed by the `GenerateCostGraphs` function within the POGG package. For the sake of simplification, the whole learning procedure dedicated to this study will be performed by the following instruction:

```
ETG_Simulations.sh
```
(3)

This script will compute set of probabilities for all transitions between each time-course sample to reproduce ammonia and nitrite quantitative variations over four years. Once computed, corresponding probabilities will be stored in modeling output files dispatched in a distinct folder called `~article/result/ammonia_nitrite/2h`. It is important to notice that the script launches several python processes that are computationally challenging on large models.

EXPORT AND ANALYSIS OF ETG MODELING RESULTS

Result export

Following the optimization process necessary to compute probabilities, pypogg package analyzes modeling results by using `read_POGG_result.py` which extracts respectively probability and sensitivity values from output files.

```
python -m scripts.read_POGG_result --help

usage: read_POGG_result.py [-h] -f file [file ...] [-osf [file]]
[-opf [file]]

optional arguments:
  -h, --help            show this help message and exit
  -f file [file ...], --result-files file [file ...]
                        result POGG files
  -osf [file], --output-sensitivity-filename [file]
                        output sensitivity filename
  -opf [file], --output-probability-filename [file]
                        output probability filename
```

```
python -m scripts.read_POGG_result -f
'ls article/result/ammonia_nitrite/2h/output_graph_ammonia_nitrite_*.txt'
```
(4)

and creates two files sensitivity.txt and probability.txt that will be further used for the sake of visualization.

Visualization

The following command produces Figure 1 in the companion manuscript:

```
python -m scripts.compute_goals
```
(5)

It writes a file `costs_ammonia_nitrite_nitrate.pkl` that is necessary to plot the evolution of ammonia, nitrite and nitrate as predicted by the probabilistic model after the training process (see Section **??**

in the manuscript).

```
python -m scripts.trajectories_simulation    (6)
```

draws probabilistic modeling results into `simulations.svg` that is Figure 1.B in the manuscript. Please note herein that

The following command:

```
python -m scripts.display_probability_sensitivity    (7)
```

produces Figure 2 as pictured in the companion manuscript. The figure depicts sets of sensitivities and probabilities between each time course by creating a svg file `probability_sensitivity.svg` that represents two heat maps; resp. the set of probabilities and sensitivities for all transitions between reactions such that the probabilistic model replicates ammonia and nitrites variations over four years.
